# Adversity Over the Life Course: A Comparison Between Women and Men Who Died by Suicide

**DOI:** 10.3389/fpsyt.2021.682637

**Published:** 2021-08-10

**Authors:** Monique Séguin, Guy Beauchamp, Charles-Édouard Notredame

**Affiliations:** ^1^Department of Psychology, University of Quebec in Outaouais, Gatineau, QC, Canada; ^2^McGill Group for Suicide Studies, Douglas Mental Health University Institute, Montreal, QC, Canada; ^3^Réseau Québécois sur le Suicide, les Troubles de l'humeur et les Troubles Associés (RQSHA), Montreal, QC, Canada; ^4^INSERM UMR1172 Lille Neurosciences et Cognition, Nord-Pas-de-Calais, Lille, France

**Keywords:** suicide, men and women comparaison, adversity over the life course, stressful events, risk factors

## Abstract

**Purpose:** This study sets out to compare the presence of life events across different domains throughout the life course which may contribute to the burden of adversity experienced differently among men and women who died by suicide.

**Method:** In a sample of 303 individuals (213 men and 90 women), data was derived from extensive clinical interviews conducted with informants. Models allowed the identification of patterns of life trajectories.

**Results:** Overall, the burden of adversity was similar across the life course except for the 5–9, 25–29, and 30–34 age ranges, where a significant difference appeared between genders [*t*-test = 2.13 (*p* < 0.05), 2.16 (*p* < 0.05) and 3.08 (*p* < 0.005), respectively] that seems to disadvantage women. The early adversities of violence and neglect, between 0 and 19 years old, are important for both groups. During the life course, women were more exposed to interpersonal adverse events such as being victims of negligence and violence, relational difficulties or abuse from their spouse, as well as tension with their own children. Men encountered more academic difficulties, legal entanglements and financial difficulties, and were more than three times more likely to develop an alcohol/drug abuse problem than women.

**Conclusions:** The data suggests some gender differences in exposure to longstanding and severe life problems contributing to suicide vulnerability. For women, the continuing burden emerges from chronic interpersonal adversities, whereas, for men, the adverse events are to a larger degree socially exposed, compounded with alcohol misuse. The adversities, especially those of a public or social nature, may be witnessed by others, which should favor the detection of vulnerability over the life course, and psychosocial or mental health services should be offered and provided earlier during the life course. Yet more men die by suicide than women. Resiliency and protective factors may benefit women to a greater degree. Future research should tackle the challenge of investigating these important elements. Meanwhile, from a public health perspective, access to psychosocial and mental health services and social acceptability of seeking services should be part of an ongoing effort in all institutional structures as a way of decreasing downstream mental health problems and vulnerability to suicide.

## Introduction

The existing literature observes significant differences in age-standardized suicide rates between genders across the life span (1–3), with lower suicide rates among females and higher suicide rates in men increasing in later life (4–6). The most important risk factors for suicide, have reached a growing scientific consensus, but have mostly been documented among males.

More recently, developmental approaches suggest that differential risk factors and adverse events could alter life courses toward suicide in different ways. Exposure to adverse childhood experiences can be either an acute event, which may be limited in duration (e.g., parental abuse or neglect), a chronic situation, which may increase the risk of other cumulative events (e.g., sexual abuse), or a long-term threat to mental health (7, 8). For example, the impact of early childhood adversity such as physical and/or sexual violence was found to be an important adverse variable, contributing to suicide vulnerability (9–12). They specifically promote the emergence of mediating variables such as aggressiveness, impulsivity, less efficient coping styles, mental disorders and, consequently, suicidal behaviors (13, 14). Differences in the types of childhood abuse have shown that women are more at risk from sexual abuse and men from physical abuse (10, 15, 16).

Developmental risk factors are associated with the complex transition that emerging adulthood (17) and midlife (18, 19) represent. Related stressful events increase the interplay between multiple roles, interrupt the achievement of major goals and may expand the feelings of distress. Therefore, gender roles may be affected differently whereas, change in status over the life course may have an impact on the access to the labor force, and leads to more women being financially independent, which has made it easier to end unsatisfying intimate relationships (20–22). Marital breakup leaves vulnerable males more exposed to solitude and to a higher suicide risk (23, 24). The increased use of social support by women (20, 25, 26), having family responsibilities (27) and being a mother (28), being pregnant (29), and raising young children (30), has been reported as protective factors for women. Other developmental risk factors, more often observed among men, include aggression, risk taking and impulsivity, which tend to peak during the period from early to mid-adulthood (31–34).

Other studies examining proximal or precipitating risk factors suggest that many stressful events are consistent with socialized gender roles, such as events that threaten one's social status or have an impact on self-esteem, identity, or well-being (35–38). Several specific events have been identified as triggers for suicidal behaviors among males, such as the end of a relationship (39), difficulties at work or the loss of a job (37), and suicide attempts (40). One of the most robustly identified proximal variables is the availability of more violent and lethal means of suicide resulting in more fatalities among men (41, 42).

### Impact of Cumulative Stressful Events Over the Life Course

The differential suicide rate among women and men is mostly explained by isolated risk or protective factors (20, 25, 26) which are rarely specific to suicide outcomes. However, integrative understandings suggest that the complex process that contributes to suicidal behavior may differ between genders (43), suggesting that men progress through the suicidal process faster than women. Explanations from social theories of role construction (28, 44) suggest that difficult transitions over the life course by experiencing loss of social status, social failures or defeat, relationship breakdowns, not achieving a conventional socially structured life or violation of expectations in regards to the timeline in which normative events should happen, tend to shape response patterns to stress. This may in turn increase suicide vulnerability (22) by threatening an individual's sense of competence.

Research methodology should take into account the impact of cumulative stress that influences risk trajectories over the life course (45). In an individual-environment interaction perspective, one must consider that exposure to stressful events may not occur at random and/or have the same impact whatever the circumstances. It is worth noting that risks or protective factors are often identified among subgroups and may not have the same benefit or harm universally (43, 46). As well, personality factors may also be the hidden causes of stressor exposure, as individuals may be more exposed to conflicts or breakdowns in relationships emerging from instability, fear of abandonment or impulsive behaviors (47, 48). Stressful events themselves may trigger a cascading effect, setting in motion other events resulting in more vulnerability to suicide (49–51). Constantly changing social and environmental factors may impact functioning and shape health disparities along gender lines (52).

The concept of allostatic load stems from biological research to explain the consequences of chronic or repeated adverse experiences by postulating the wear and tear of stress-regulatory mechanisms (45). Allostatic load may lead to illness (53) and compromises health, not only because of the stress experiences themselves, but also because of damaging behaviors and maladaptive coping strategies that frequently accompany chronic stress states (53). Research on the association between stressful life events and health (7) showed empirical substantiation for the role of stressors in disease risk and ultimately on suicide vulnerability.

By considering the consequences of cumulative adverse events under the notion of burden of adversity, this paper aims to identify the differences in life courses toward suicide between men and women.

## Methods

### Participant Recruitment

Through an ongoing partnership with the Quebec coroner's office, our research group is constantly recruiting family members of individuals who recently died by suicide in the province of Quebec (Canada). We report on 303 suicide cases: 213 men and 90 women. Data was retrieved from participants recruited in the provinces of Québec and New Brunswick, Canada, across four different research projects conducted between 2003 and 2012. In each project, the protocol was established as follows: after the family received an introductory letter from the coroner's office, a research assistant followed up with a telephone call (~70% of the close relatives referred by the coroner's office agreed to participate in the study), and, if participants agreed to participate, an appointment was made for the interview. This successful partnership has enabled our research group to pursue recruitment of suicide cases for all these studies.

### Procedure for Data Collection

The interviews occurred between 6 and 18 months after the death, when written informed consent was signed. Skilled investigators conducted two in-depth interviews with each informant which lasted 2 to 3 h on average and comprised three sections: exploration of socio-demographic characteristics; psychopathological investigation with the administration of the SCID I (54) and SCID II (55); and inventory of adverse life events [life trajectory calendar method (56)]. Personal written documents belonging to the deceased and the informants, such as photos, agendas and diaries, were also used if available as memory triggers during the interview. After the interview process, medical and psychosocial files of the deceased were also examined to corroborate the information on the presence of adversity and mental health diagnoses during specific periods of life. These medical and psychosocial reports were obtained upon signed agreement of family members. Afterwards, a case vignette containing a summary of all clinical information was drafted and submitted to a panel of experts. This panel established a consensus rating every 5 years throughout the life course in regards to a summary variable identified as “burden of adversity” ranging in a six-point scale from severe to low adversity.

The protocol received approval from the ethics review boards of the Douglas Mental Health University Institute and the University of Québec in Outaouais (Nos. 2,362; 2,533; 2,608; 2,856). All informants signed a consent form.

### Measurements

#### Interview to Determine Post-mortem Diagnosis

The post-mortem diagnoses were assessed using a validated follow-back method (57, 58), using a semi-structured questionnaire, the Structured Clinical Interview for DSM-IV, for both Axis I and Axis II disorders (SCID I and II) (54, 55), with an informant who had known the deceased well. This procedure has been described in other papers (59, 60). A series of studies over the past decade have established the concordance of DSM diagnoses generated by informant report with chart diagnoses and the psychological autopsy method have been proven with good reliability (58, 61, 62). Categorical inter-rater reliability revealed moderate to excellent inter-rater agreement of the Axis I disorder, while most categorically and dimensionally measured personality disorders showed excellent inter-rater agreement (63).

#### Interview to Retrace the Life Trajectory

To collect all the possible life events that the participants had encountered, we carried out semi-structured conversational explorations inspired from life calendar narrative methods (64–66). The interviews explored events occurring during the life course and type of events occurring in different qualitative spheres of life. During the interviews, a “life calendar” explored nine clearly described conceptual spheres: parent-child relationship and early adversities; affective life sphere; procreation and family life; relationship with siblings and extended family; academic difficulties; professional difficulties; social life and relational difficulties; living conditions; and losses/personal adversity such as legal and financial difficulties. For each sphere events possibly occurring were investigated. For example, in the sphere of parent-child relationship and early adversities, events such as maltreatment, physical, and sexual abuse, negligence, tension in parent child relation were investigated. In the affective life sphere, relational difficulties with spouse such as violence, multiple breakups, etc., were investigated.

The method explores major events which may occur at a specific age for each sphere and assesses the severity and duration of each event chronologically. The integration of a visual calendar helps with the recall of events (the horizontal axis represents the passing of time and the vertical axis, the specific spheres in which events occurred). Participants were also encouraged to access other visual aids to help them recall events during the interview process.

The full list of events was inspired from widely accepted comprehensive interview guides, such as the Life History Calendar (67) or the or the Childhood Experience of Care and Abuse (CECA) (68), which we completed from a scoping review of the main self-administered life adversity questionnaires. Based on our extensive practice of narrative explorations, the resulting adversity statements were refined to minimize overlaps between items while ensuring the broadest coverage of possible significant stressful experiences.

After the interviews, clinical case histories (case vignettes) were drafted. The vignettes took into account information from the socio-demographic questionnaire, the SCID I and II, the information from hospital files and information gathered with the life trajectory questionnaire. While narrative methodology is largely used qualitatively, it can also be used in quantitative and mixed methods studies (69) with data transformation.

### Data Transformation Into a Score of Burden of Adversity

Qualitative data collected from the narrative interviews was transformed into a summary variable identified as “burden of adversity.” This summary variable was built to reflect the “contextual threat” weighting on the individual, which is based on the morbidity burden or low disease burden approach (70, 71) used to identify the overall morbidity that affects health. It is also related to the concept of allostatic load, which links psychosocial stress (with the neurobiological and genetic dimensions) and its impact on mental disorders and suicide (45, 69).

To estimate the level of burden of adversity, a panel of clinicians and researchers analyzed the life trajectory vignettes and evaluated the relative adversity weight within the respondent's developmental circumstances. Based on this evaluation, experts gave, for each 5-year interval, an overall burden of adversity rating ranging from low (rating of 1 or 2) to moderate (3 or 4) to severe (5 or 6). For example, a severe rating (5 or 6) would entail sustaining major adversity, such as being a victim of physical and sexual violence persistently and having other constant adversities in other spheres of life during the same 5-year period. A low rating (1 or 2) would be characterized as having occurrences of adversities confined to one or two spheres of life, while sustaining protective factors in other spheres. Case reference logs were written and used to maintain the same level of evaluation across all cases. In each case, the evaluators independently coded each 5-year period before reaching a consensus through discussion. In studies from our group, the intra-pair agreement for each 5-year segment ranged from 97 to 76%, where the lowest intra-group agreement was in the 0 to 4-year age group (56). This methodology has previously been presented in other papers in great detail (59).

### Analytical Strategy

To examine the characteristics obtained from the life trajectory questionnaire that distinguish gender along gender lines, we grouped questionnaire items into themes, such as being a victim of early violence, negligence, intimate relationship difficulties, academic difficulties, social difficulties, etc., which were themselves representative of the different spheres. These thematic characteristics were simply scored as present or absent (dichotomous variables). A series of contingency table analyses were conducted to determine the association of each individual theme separately in relation to the gender. Effect size was given as odd ratios (OR).

Burden of adversity trajectory data were examined *via* latent growth curve analysis (LGCA) in MPLUS based on the structural equation modeling framework with gender as the group variable. In testing growth models with discrete time survival analysis (DTSA) (joint model, taking into account the time-dependent risk of dying) or without DTSA, quality of the different models was estimated by the following information criteria: Akaike (AIC) and Bayesian (BIC). Lower AIC and BIC values indicate a better-fit model. A binary covariate (deceased/living) appeared in the joint model only, for every time point in the DTSA part. There was no covariate in the growth model without DTSA. The latent growth parameters intercept, linear and quadratic terms of women and men were compared by Z-score of differences. Specific age-period data points were compared by a *t*-test. Socio-demographic data was analyzed by *t*-test or contingency tables where appropriate and Chi square values given. Survival Analysis was determined by Kaplan-Meier curves and confidence intervals at each age period. Furthermore, a Chi square logrank calculation was applied to compare gender survival curves.

## Results

### Socio-Demographic and Survival Data

**Figure 1:**
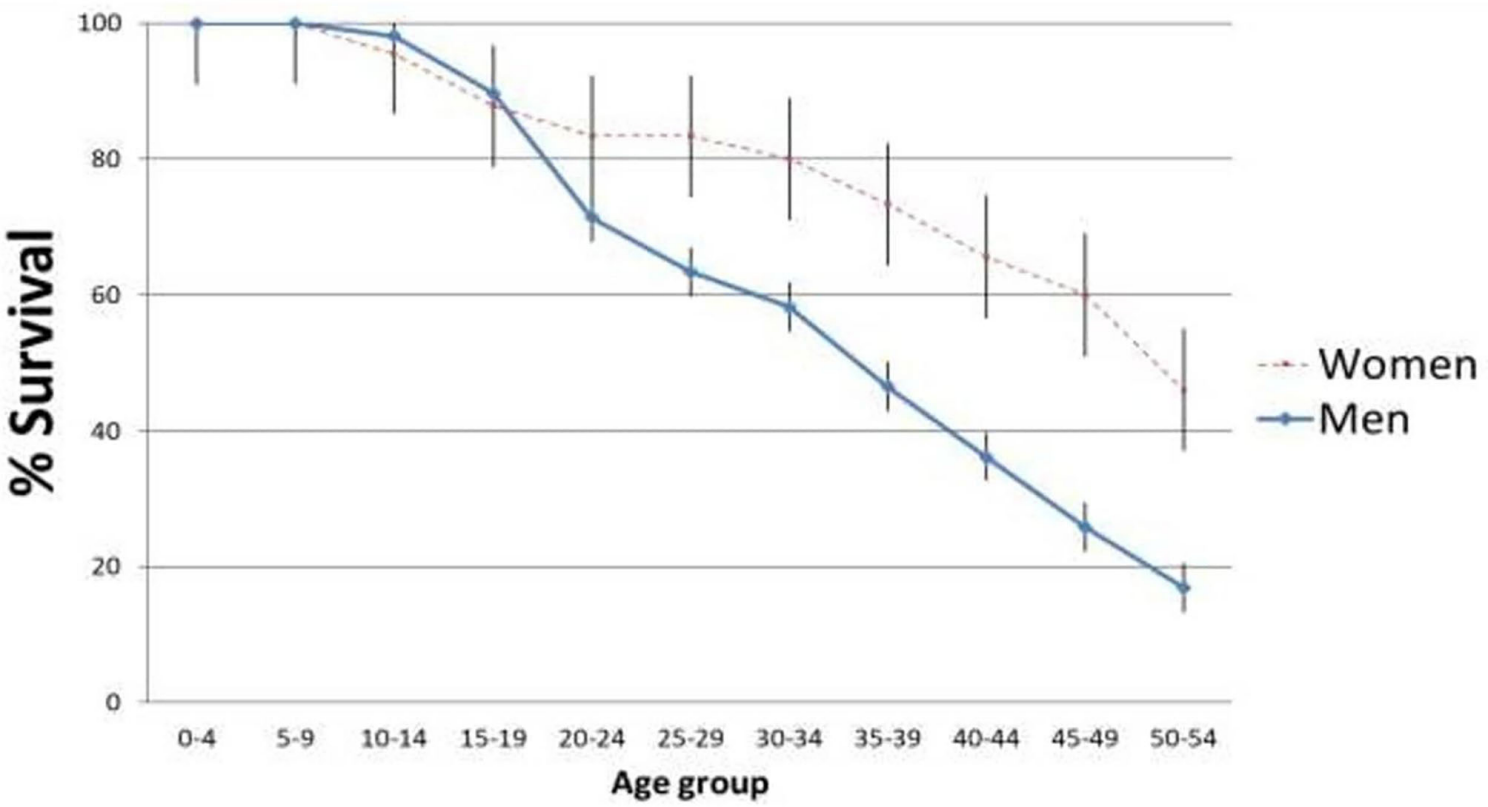
Survival analysis of groups in regards to the occurrence of death. Vertical bars corresponds to standard errors.

Among the 303 suicide cases, 70% were men, with a mean age at the time of death for women was 45.5 (SD = 17.3), and that of men was 38.4 (SD = 15.4). Survival analysis (Figure 1) clearly indicates an earlier occurrence of death in men and a lower median survival time (30–39 vs. 40–49). Kaplan-Meier curves and confidence intervals of each time point are represented in Figure 1. A logrank analysis revealed a significant Chi-square value of 10.17 (*p* < 0.005), indicating a sharp difference in pattern for gender. All points beyond the 15–19 age period was significantly different for men and women and there was some form of parallelism in the curve shapes after 24 years of age.

Based on the socio-demographic information (Table 1), at the time of death, men and women had a similar profile with respect to marital status and paid work habits. Differences appeared in other areas of comparison. Although, approximately half of the subjects (both genders) had no children, a higher percentage of women had two or more children compared to men (42.2 vs. 28.2%; χ^2^ = 5.71, *p* < 0.05). A greater proportion of men were self-supported (45.5 vs. 28.8%; χ^2^ = 7.26, *p* < 0.05), while a greater proportion of women were State-supported (25.6 vs. 8.9%; χ^2^ = 14.66, *p* < 0.001) and had obtained a University-level degree (22.2 vs. 10.8%; χ^2^ = 6.78, *p* < 0.01).

**Table 1:** Socio demographic characteristics.

	**Women** ***n*** **= 90**	**Men** ***n*** **= 213**	**Ch^2^**	***P*** **-value**
**Marital status**				
Married	28 (31.1%)	63 (29.6%)	0.07	NS
Dating	35 (38.9%)	99 (46.5%)	1.48	NS
Separated/divorced/ widow(er)	27 (30.0%)	49 (23.0%)	2.65	NS
Unknown	0 (0%)	2 (0.9%)		
**Number of children**				
0	39 (43.3%)	111 (52.1%)	1.95	NS
1	13 (14.5%)	39 (18.3%)	0.66	NS
2+	38 (42.2%)	60 (28.2%)	5.71	<0.05
Unknown	—	3 (1.4%)		NS
**Education**				
High School not completed	2 (2.2%)	17 (8.0%)	3.57	NS
High School	52 (57.8%)	140 (65.7%)	1.72	NS
Collegey	16 (17.8%)	33 (15.5%)	0.24	NS
University	20 (22.2%)	23 (10.8%)	6.78	<0.01
**Income**				
Self-supported	26 (28.8%)	97 (45.5%)	7.26	<0.01
From family member	18 (20.0%)	51 (23.9%)	0.56	NS
From State	23 (25.6%)	19 (8.9%)	14.66	<0.01
Unknown/missing	23 (25.6%)	46 (21.6%)		
**Living arrangements**				
Lives alone	37 (41.1%)	65 (30.5%)	3.18	NS
Lives with a family member	29 (32.2%)	89 (41.8%)	2.43	NS
Shares with room-mate/transition home	5 (5.6%)	17 (8.0%)	0.55	NS
Other	19 (21.1%)	42 (19.7%)		
**Paid work**				
Yes	48 (53.3%)	119 (55.9%)	0.16	NS
No	31 (34.4%)	76 (35.7%)	0.04	NS
Unknown	11 (12.2%)	18 (8.5%)	1.04	NS

### Mental Health Data

Table 2 compares life and active psychiatric diagnoses between men and women. Although, the proportion of individuals with anxiety, affective and psychotic disorders was similar between genders, alcohol-related disorders were more prominent in males (life: 52.1 vs. 30.0%, χ^2^ = 12.47, *p* < 0.001; active: 45.1 vs. 18.9%, χ^2^ = 18.54, *p* < 0.001). Men were also more likely to have two or more Axis I disorder (54.5 vs. 26.7%, χ^2^ = 19.66, *p* < 0.001), a conduct disorder (12.7 vs. 2.2%, χ^2^ = 7.99, *p* < 0.01) and two or more Axis II disorders (23.5 vs. 10.0%, χ^2^ = 7.32, *p* < 0.01) than women.

**Table 2:** Presence of psychiatric diagnoses current and life course.

**Disorder**	**Men (213)**		**Women (90)**		**Chi^2^**	***P*** **-value**
	***N***	**%**	***N***	**%**		
Anxiety (life)	31	14.6	14	15.6	0.05	NS
Anxiety (active)	23	10.8	8	8.9	0.25	NS
Affective (life)	87	40.8	38	42.2	0.05	NS
Affective (active)	137	64.3	55	61.1	0.28	NS
Psychotic (Life)	15	7	6	6.7	0.01	NS
Psychotic (active)	16	7.5	6	6.7	0.07	NS
Alcohol (life)	111	52.1	27	30	12.47	<0.001
Alcohol (active)	96	45.1	17	18.9	18.54	<0.001
Conduct disorder (before 18 years of age)	27	12.7	2	2.2	7.99	<0.01
0 Axis I diagnosis	23	10.8	13	14.4	0.8	NS
1 Axis I diagnosis	64	30	45	50	10.94	NS
2+ Axis I diagnoses	116	54.5	24	26.7	19.66	<0.001
Unknown	10	4.7	8	8.9	1.99	NS
Personality disorder	87	40.8	27	30	3.17	NS
0 Axis II diagnosis	83	39	26	28.9	2.79	NS
1 Axis II diagnosis	53	24.9	28	31.1	1.25	NS
2+ Axis II diagnoses	50	23.5	9	10	7.32	<0.01
Unknown	27	12.7	27	30		

### Life Events and Burden of Adversity Data

When considering life events, men and women differed on certain aspects of specific life spheres (Table 3). Results indicate that both groups suffered important adversity between the age of 0 and 19 years old, such as sexual/physical or psychological violence. Ranging between 19 and 37% for women, increasing from ages 5 to 14, then decreasing slightly. For men, the sexual/physical or psychological violence ranged between 22.5 and 27% over the course of different age periods from 0 to 19 years old. Another important adversity is the presence of discipline/neglect tension in the relationship with parents, ranging from 50 to 76% for women and from 47 to 69% for men. Although, there was a decrease in neglect and violence in adult years, women were more prone to be victims of neglect in the 25–29 age group (ORs significantly lower than 1). During youth, men encountered more academic difficulties than women (ages 10–14, 32.4 vs. 17.8%), and were more prone to legal entanglements (ages 15–19, 16.5 vs. 5.8%). Women are approximately three times more likely to encounter relational difficulties with their spouse until 44 years of age.

**Table 3:** Life events: comparison between man and women.

**Life events** ***N*** **= 303**	**Men** **(***n*** = 213)**		**Women** **(***n*** = 90)**		**OR**	**CI**_**95%**_	***p***
	***n***	**%**	***n***	**%**			
Age 0–4							
Discipline/neglect/ tension in parent-child relationship	101	47.4	45	50.0	1.05	0.686–1.620	NS
Sexual abuse/physical- psychological violence of S	48	22.5	17	18.9	0.84	0.457–1.536	NS
Age 5–9	(*n =* 213)		(*n =* 90)				
Discipline/neglect/ tension in parent-child relationship	117	54.9	58	64.4	1.17	0.787–1.750	NS
Sexual abuse/physical- psychological violence of S	57	26.8	28	31.1	1.16	0.694–1.950	NS
Academic difficulties	46	21.6	14	15.60	0.72	0.377–1.376	NS
Relational difficulties	0	0	0	0	–	–	NS
Age 10–14	(*n =* 213)		(*n =* 90)				
Discipline/neglect/ tension in parent-child relationship	129	60.6	68	75.6	1.25	0.850–1.830	NS
Sexual abuse/physical- psychological violence of S	52	24.4	33	36.7	1.5	0.910–2.479	NS
Mental health problems	46	21.6	18	20.0	0.93	0.505–1.684	NS
Academic difficulties	69	32.4	16	17.8	0.55	0.302–0.997	<0.05
Relational difficulties	7	3.3	4	4.4	1.35	0.386–4.734	NS
Legal difficulties	12	5.6	2	2.2	0.39	0.087–1.798	NS
Age 15–19	(*n =* 212)		(*n =* 86)				
Discipline/neglect/ tension in parent-child relationship	125	59.0	60	69.8	1.18	0.796–1.760	NS
Sexual abuse/physical- psychological violence of S	49	23.1	29	33.7	1.46	0.865–2.462	NS
Mental health problems	72	34.0	20	23.3	0.68	0.393–1.193	NS
Academic difficulties	58	27.4	15	17.4	0.64	0.343–1.186	NS
Professionnal difficulties	15	7.1	3	3.5	0.49	0.139–1.746	NS
Relational difficulties	44	20.8	25	29.1	1.4	0.807–2.430	NS
Financial difficulties	23	10.8	3	3.5	0.32	0.094–1.100	NS
Legal difficulties	35	16.5	5	5.8	0.35	0.133–0.929	<0.05
Age 20–24	(*n =* 191)		(*n =* 79)				
Discipline/neglect/ tension in parent-child relationship	64	33.5	30	38.0	1.13	0.683–1.881	NS
Mental health problems	121	63.4	48	60.8	0.96	0.627–1.467	NS
Professionnal difficulties	21	11.0	7	8.9	0.81	0.329–1.972	NS
Relational difficulties	62	32.5	25	31.6	0.97	0.572–1.662	NS
Relational difficulties with spouse	22	11.5	25	31.6	2.75	1.463–5.159	<0.005
Financial difficulties	33	17.3	8	10.1	0.59	0.259–1.325	NS
Legal difficulties	35	18.3	53	3.8	0.21	0.062–0.693	<0.01
Age 25–29	(*n =* 152)		(*n =* 75)				
Discipline/neglect/ tension in parent-child relationship	25	16.4	26	34.7	2.11	1.140–3.900	<0.05
Mental health problems	87	56.2	45	60.0	1.05	0.666–1.650	NS
Professionnal difficulties	17	11.2	12	16.0	1.43	0.650–3.150	NS
Relational difficulties	35	23.0	18	24.0	1.04	0.554–1.961	NS
Relational difficulties with spouse	24	15.8	24	32.0	2.03	1.080–3.804	<0.05
Financial difficulties	24	15.8	9	12.0	0.76	0.336–1.716	NS
Legal difficulties	30	19.7	9	4.0	0.2	0.060–0.686	<0.02
Age 30–34	(*n =* 135)		(*n =* 72)				
Mental health problems	78	57.8	45	60.5	1.08	0.679–1.723	NS
Professionnal difficulties	19	14.1	8	11.1	0.79	0.329–1.892	MS
Relational difficulties	23	17.0	14	19.4	1.14	0.354–2.352	NS
Relational difficulties with spouse	22	16.3	27	37.5	2.3	1.223–4.327	<0.01
Financial difficulties	29	21.5	12	16.7	0.78	0.374–1.612	NS
Legal difficulties	19	14.1	2	2.8	0.2	0.045–0.871	<0.05
Age 35–39	(*n =* 124)		(*n =* 67)				
Mental health problems	79	63.7	45	67.2	1.05	0.658–1.690	NS
Professionnal difficulties	17	13.7	11	16.4	1.2	0.530–2.704	NS
Relational difficulties	29	23.4	14	20.9	0.89	0.442–1.810	NS
Relational difficulties with spouse	25	20.2	30	44.8	2.22	1.209–4.080	<0.01
Financial difficulties	32	25.8	14	20.9	0.81	0.404–1.622	NS
Legal difficulties	22	17.7	8	11.9	0.67	0.284–1.594	NS
Age 40–44	(*n =* 99)		(*n =* 62)				
Mental health problems	64	64.6	42	71.2	1.1	0.664–1.825	NS
Professionnal difficulties	16	16.2	10	16.9	1.05	0.447–2.462	NS
Relational difficulties	22	22.2	16	27.1	1.22	0.594–2.510	NS
Relational difficulties with spouse	18	18.2	24	40.7	2.24	1.121–4.465	<0.05
Financial difficulties	35	35.4	8	13.6	0.38	0.167–0.882	<0.05
Legal difficulties	14	14.1	4	6.8	0.48	0.151–1.525	NS
Age 45–49	(*n =* 77)		(*n =* 54)				
Mental health problems	60	77.9	42	77.8	1	0.590–1.689	NS
Professionnal difficulties	13	16.9	7	13.0	0.77	0.287–2.051	NS
Relational difficulties	16	20.8	15	27.8	1.34	0.609–2.932	NS
Relational difficulties with spouse	16	20.8	16	29.6	1.43	0.657–3.096	NS
Financial difficulties	25	32.5	11	20.4	0.63	0.285–1.382	NS
Legal difficulties	11	14.3	4	7.4	0.52	0.157–1.715	NS
Age 50–54	(*n =* 55)		(*n =* 42)				
Mental health problems	41	74.5	35	83.3	1.12	0.611–2.045	NS
Professionnal difficulties	12	21.8	4	9.5	0.44	0.131–1.450	NS
Relational difficulties	8	14.5	5	11.9	0.82	0.250–2.683	NS
Relational difficulties with spouse	13	23.6	10	23.8	1.01	0.403–2.520	NS
Financial difficulties	18	32.7	9	21.4	0.65	0.267–1.603	NS
Legal difficulties	10	18.2	6	14.3	0.79	0.264–2.334	NS

*Parent-child relationship and early adversities we regrouped specific events into two categorie*.

*(i) problems with discipline/neglect/tension in the parent child relationship including incoherent rules, lack of discipline, role reversal, affective distance, parental mental health difficulties, witnessing parental violence*.

*(ii) sexual abuse/physical and psychological violence*.

*Mental health problems included variables such as Axis I diagnosis, personality disorder, alcohol and drug abuse, number of suicide attempts, other problems of mental health, and psychiatric hospitalization*.

Relational difficulties with spouse include psychological, physical and sexual violence, breakups, infidelity, jealousy, control over one partner, entrapment, etc.

*Relational difficulties included variables such as conflict with peers, social isolation, bad influence from peers, separation or loss of a friend, etc*.

*Academic difficulties included events such as: underachievement, failure in school, behavioral problems in school, being a bully, etc*.

*Legal problems included all types of behaviors resulting in negative events with the justice system*.

*Financial losses included bankruptcy, financial hardships etc*.

At ages 25–29, women incur more discipline, neglect and tension with their own children, in parent-child relationships, than men (34.7 vs. 16.4%). As adults, men continue to have greater legal difficulties until 35 years of age. During the period between 40 and 44 years old, one third of men experience financial difficulties.

**Figure 2:**
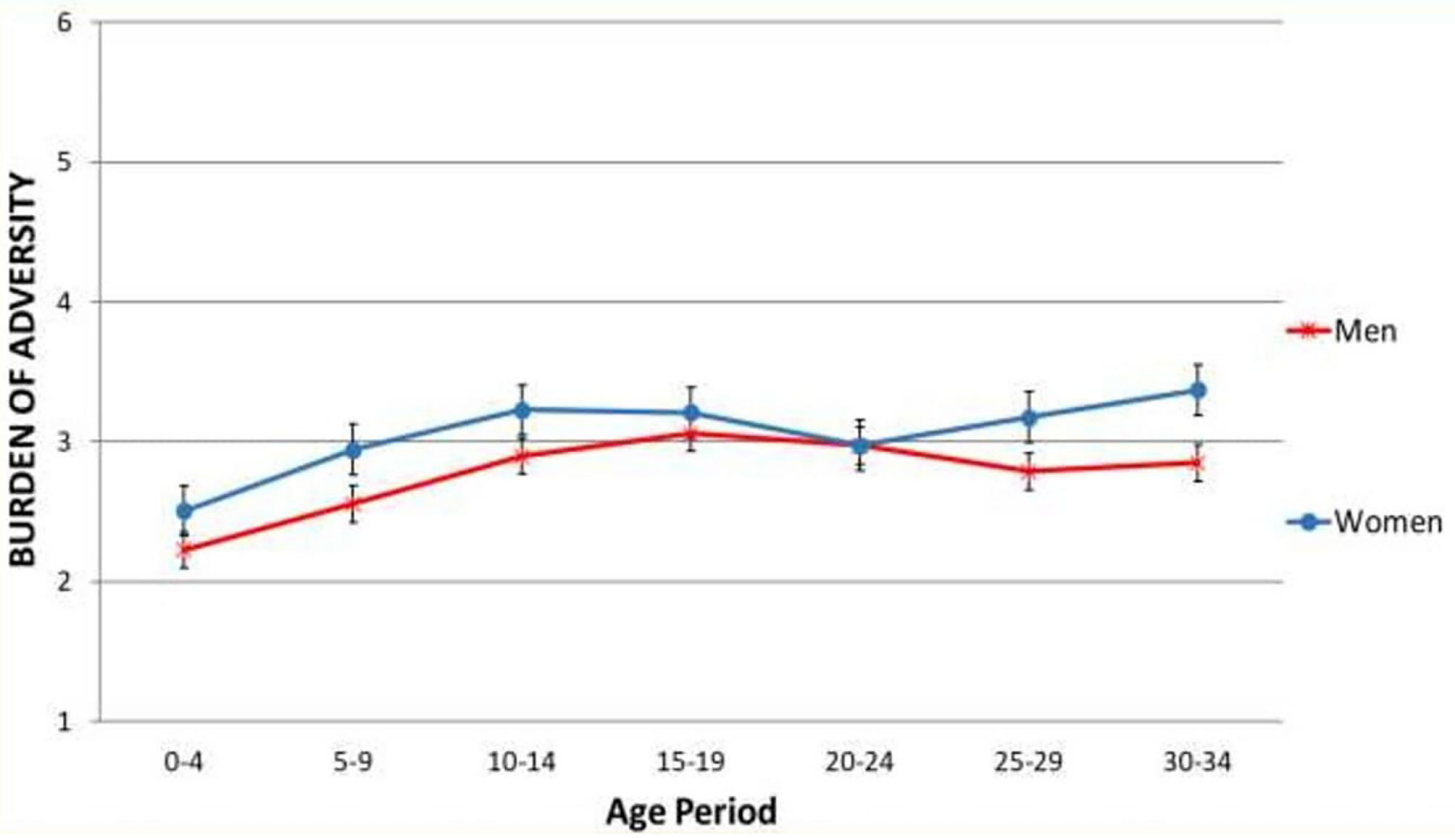
Growth model analysis representing the burden of adversity trajectories. Vertical bars corresponds to standard errors.

In testing growth models with or without discrete time survival analyses in MPLUS, the model without discrete time survival analysis showed lower values of AIC (5130.280 vs. 5629.895) and BIC (5204.555 vs. 5719.025), so the model without was adopted. On examining the burden of adversity trajectories, both genders exhibited significant gradual increases over age periods (Figure 2, Table 4). Overall, the burden of adversity was similar except for the 5–9, 25–29 and 30–34 age periods, where a significant difference appeared between genders [*t*-test = 2.13 (*p* < 0.05), 2.16 (*p* < 0.05), and 3.08 (*p* < 0.005), respectively]. Growth model parameter analysis (Table 2) results indicate that a significant intercept, linear, and quadratic terms exist in both genders' trajectory, revealing a V-shape deviation in the burden of adversity values in mid age periods. On comparing the different term values of genders, no significant z-values appeared (Z = 1.56, −0.30, and 0.16 for intercept, linear, and quadratic terms, respectively).

**Table 4:** Burden of Adversity trajectories curve parameters.

**Parameters (SE)**	**Men**	**Women**
Intercept	2.224 (0.089)^a^	2.586 (0.169)^a^
Linear change	0.347 (0.042)^a^	0.275 (0.075)^a^
Quadratic change	−0.026 (0.007)^a^	−0.019 (0.011)

a *p < 0.001*.

## Discussion

### Socio-Economic Disparities

Survival analysis shows that men have a lower life expectancy than women. Compared to men, more women report living alone, having lower income and being financially supported by State income, even if more women attain a higher level of education. Results of our study indicate that almost half of the women (43%) and more than half of the men (52%) did not have children, while 42% of those mothers and 28% of fathers had two children or more. Research at the population level indicates that parenthood among 25 to 44*-*year*-*olds is associated with a lower suicide risk in both men and women, but to a larger extent among women, and particularly in parents with two or more children (72). However, for vulnerable individuals, having children may not always be protective, and other cumulative events may impact on their feeling of being a burden to their families.

The importance of socio-demographic factors in suicide research, except for age and gender, is often overlooked. In the present study, women seem to be at a disadvantage regarding some socio-demographic variables (such as work, income, living alone, parental responsibilities). Socio-demographic variables such as education, access to well-paying jobs and access to daycare for children are important determinants of health (73). Conversely, research indicates that early socioeconomic adversity contributes to poor mental health trajectories, disrupt successful transition into adulthood, and endangers social, academic, and occupational attainment (74). Several studies do make the case for socio-demographic disparities between genders (75) which contribute to the overall burden of adversity by increasing the probability of cumulative risk factors. In this study, while women have more risk factors in regards to socio-demographic variables, this fact doesn't translate into mental health disparities, when compared to what is observed among men.

### Mental Disorder Differences

Our results didn't show inter-gender differences in depressive disorders. However, men display overall more frequent mental disorders than women and are three times more likely to have alcohol/drug abuse problems. In early life, they also exhibited more frequent conduct disorders, which may signal differential psychopathological pathways toward suicide between men and women with more internalizing of problems in girls and externalizing of problems in boys (33). However, it is important to note the ongoing debate as to the prevalence, and lack of appropriate gender criteria for conduct behavior may lead to underreporting conduct disorders in females.

Results also indicate that men are more prone to alcohol misuse during the life course and in the months prior to death. Researchers found alcohol abuse and drug use to be the most common factor among suicide cases (76). Alcohol abuse may generate a succession of events including professional and romantic failures, difficulties in social relationships, distress and mental health problems; trigger adverse life events; and progressively lead to an earlier exhaustion of adaptative mechanisms. Compounded with early mental health difficulties (such as conduct disorders), men display, during their adult life, more comorbid psychiatric disorders than women. The presence of early externalized conduct (32), and an ongoing abuse of drugs/alcohol, is seen as a distal and proximal risk factor for suicide (46). Resulting interpersonal difficulties and social isolation further fuel the spiral of adversity.

A prior history of suicide attempts is considered one of the most robust predictors of completed suicide. The presence of one or more suicide attempts is an important factor for subsequent death (77). In a Swedish study, researchers found a mean of 3.5 suicide attempts before the eventual suicide (78). Coherent with these observations, our data indicates that 45% of the men and 28% of the women had attempted suicide at least once in their life course.

### Stressful Events and Burden of Adversity

The data indicates that both groups suffered important early adversity between the ages of 0 and 19 years old. Although, both genders were exposed to longstanding and severe life problems, likely to have contributed to suicide vulnerability, the nature of this adversity differed between men and women. For women, the parent/child relationship adversities evolved in some way into interpersonal adversities with their marital partner/spouse and, later on, into difficulties in relationships with children. As for men, the adverse events are to a larger degree socially exposed events such as academic difficulties (10–14 years old), downstream legal difficulties (between 15 and 34 years old) and financial difficulties (between 40 and 44 years old) compounded with alcohol misuse. From a social perspective, women who are victims of violence experience myriad devastating consequences in regards to health, well-being, quality of life, and impact on their participation and engagement in society at large (79).

Results indicate that life trajectories between men and women differ by the number and nature of adverse events, that burden of adversity over the life course is slightly higher for women at almost all periods of life, except for a merging period around the early twenties. The burden of adversity remains low to moderate (score of 2 to 3) throughout the life course. A number of participants did experience severe adversity, having lived through extreme and traumatic events at some period of their lives. When aggregating all scores of “total burden of adversity” in order to obtain a comparison between groups, we lose the intra-group differences.

Suicide vulnerability is explained through complex mechanisms and interactions and a number of developmental risk factors lead to the presence of mental disorders and adverse life events as proximal factors leading to the suicidal outcome. However, the complexities of theses mechanisms (impulsivity traits, attachment, internalization vs. externalization, emotional regulation, etc.), may be difficult to observe or confirm, especially from retrospective research with informants. In the present research, those developmental mechanisms were not evaluated, but we may suspect the presence of an internalization or externalization mechanism which could be accounted by the specific types of adverse events occurring among men and women. We found men to have more alcohol, conduct, and personality disorder compared to women and men were more prone to negative academic, legal, financial problems compared to women. Other published data indicate that externalizing spectrum disorders (ESDs), are known to be associated to academic underachievement, underemployment, criminal behavior, incarceration, and other adverse outcomes (80). In comparison, the presence of interpersonal difficulties observed among women, may indicate the presence of internalization behaviors from an early age. A longitudinal study in a community sample of mistreated adolescents found that internalizing symptoms were predictive of dating aggression (81). The link between symptoms of distress and internalizing symptoms among females may be a gender specific consequence of an inability to effectively cope with affective distress (82). Data from other research (83), have shown that child abuse is associated with either internalizing or externalizing behaviors for both males and females. The impact of these early adversities may put into motion complex developmental mechanisms, along the lines of internalization or externalization behaviors which may have a gendered specific component and may generate different types of negative events across the life cycle.

These findings suggest that gender is an important variable to explore, since the lives of women and men are characterized by different trajectories of adversities (74) and a higher rate of suicide mortality among men. Future research should tackle the challenge of collecting and analyzing a broad range of measures from biological to psychosocial dimension at multiple time points in order to better understand the mechanisms by which certain individuals may be more resistant to stressful live adversity (75).

### Limitations and Methodological Considerations

Firstly, this sample may not be representative of all suicide cases due to a selection bias. However, to our knowledge, it's the largest sample of suicide for which in-depth collection of adverse life events has been carried out so far.

Secondly, in this paper, we proposed to examine an aggregated pathway for women and men in order to compare the main difference between the groups, which may have masked intra-group differences. Eventually, research should concentrate on divergent pathways and accompanying growth models, which would capture more diversified developmental stages.

Thirdly, using a follow-back and life calendar method to assess the presence of mental health symptoms and life events by third parties entails recall biases or imprecise information and underreporting (84). Recall biases when collecting data through proxy-based, retrospective explorations of suicide trajectories have been frequently discussed in the literature (85). Informants may remember more easily mental health symptoms or events that are observable, e.g., externalized behaviors vs. internalized behaviors. Even good informants may not have been aware of a number of mental health symptoms or personal adverse events. By definition, life description will mostly be based on publicly known events. As well, the events reported by informants are usually marked or moderate in intensity, which is why they are able to remember specific events. Another possible bias may have been introduced by not concealing decedent gender form panel. The severity of some events may have been perceived differently in regards to gender, but the overall adversity score for each 5 years period were based on a coding book based on the presence of types of adversity their numbers and duration over the 5-year period.

While keeping these limitations in mind, numerous authors suggest that narrative-rating instruments provide large gains in reliability and validity in the measurement of major stressful events (50, 65). Lin et al. (86) indicate that the recall error usually reflected underreporting rather than overreporting. In previous publications, we provided detailed descriptions of the measure we took to minimize these biases (59, 87). In brief, we used semi-structured and conversational-style interviews, searched for pre-specified adverse life events, used memory anchors, stimulated recall efforts with calendars and photos and cross-checked collected data from various sources. However, the possibility of remaining reporting filters and omissions cannot be completely discounted.

## Conclusion

Results indicate the importance of recognizing differential adverse events in the lives of women and men which develops onto longstanding and severe problems creating difficulties in relating to close relationships and society in large. From a public health perspective, it is important to better identify the severe adverse events occurring during key developmental periods, from childhood (being a victim of violence and neglect), to adolescence (academic difficulties, social isolation), to adulthood (misuse of alcohol/drugs, mental health problems, violence, interpersonal difficulties), and implement specific targeted preventive strategies. Results from this study suggest that the nature of the events during adolescence and adulthood should favor the detection of vulnerability, especially when the adversities are of public nature. These adversities, especially if they are repetitive, may be witnessed by others over the life course, and psychosocial or mental health services should be offered and provided. From a public health perspective, access to psychosocial and mental health services and, more specifically, addressing the social acceptability of seeking these services should be part of an ongoing effort in all institutional structures (schools, work places, households) and should be “tucked in” every health strategy, as a way of decreasing downstream mental health problems and vulnerability to suicide.

## Data Availability Statement

The raw data supporting the conclusions of this article will be made available by the authors, without undue reservation.

## Ethics Statement

The studies involving human participants were reviewed and approved by Douglas Mental Health University Institute and the University of Québec in Outaouais (Nos. 2362; 2533; 2608; and 2856). All informants signed a consent form. The patients/participants provided their written informed consent to participate in this study.

## Author Contributions

MS conceived the original idea, conducted the literature review, set the conceptual basis of the model, collected the data, and lead the writing of the manuscript. GB did the statistical analyses and helped with the writing of the manuscript. C-ÉN reviewed the manuscript. All authors contributed to the article and approved the submitted version.

## Conflict of Interest

The authors declare that the research was conducted in the absence of any commercial or financial relationships that could be construed as a potential conflict of interest.

## Publisher's Note

All claims expressed in this article are solely those of the authors and do not necessarily represent those of their affiliated organizations, or those of the publisher, the editors and the reviewers. Any product that may be evaluated in this article, or claim that may be made by its manufacturer, is not guaranteed or endorsed by the publisher.
